# Supplementary material for: Cancer-associated fibroblast spatial heterogeneity and EMILIN1 expression in the tumor microenvironment modulate TGF-β activity and CD8+ T-cell infiltration in breast cancer
Source: Theranostics. 2024 Feb 24;14(5):1873–85. doi: 10.7150/thno.90627 (PMC10945331; doi:10.7150/thno.90627)
Supplement: Supplementary file 1 — Supplementary figures and tables. [file thnov14p1873s1.pdf]

SUPPLEMENTARY TABLES AND FIGURES

Table S1: Clinical and pathological information on the patients whose BC samples (A1, B1, C1 and D1) were used for the spatial analysis.

Table S2: Clinical and pathological information on the patients whose BC samples (N=75) were used for the validation analysis.

Table S3: Gene expression signatures of the individual cell populations used in the present study.

Table S4: The relationship between EMILIN1 expression and clinico-pathological variables in the cohort of 75 patients with BC. P-values were calculated using the $\chi 2$ test.

Figure S1: Epithelial and fibroblast populations in the newly generated BC single-cell atlas. (**A**) InferCNV analysis of epithelial cancer cells. (**B**) Gene expression analysis of individual CAF subpopulations.

Figure S2: Analysis of individual CAF subpopulation gene expression signatures (**Table S3**) in CAF cells from (**A**) Kieffer et al. and (**B**) Wu et al. datasets.

Figure S3-S12: Spatial distribution of different cellular populations in BC samples (patients A1-D1, present study; patients 1160920F, 1124423F, CID4290, CID44971, CID4465 and CID4535 from Wu et al. dataset). Labels TGFb, IFNab and IFNg refer to TGFβ, IFNαβ and IFNγ respectively.

Figure S13: Spatial distribution of selected GO processes in the remaining eight BC samples. Shown are the following GO processes: ECM Structural Organization, Wound Healing, TGF-β Receptor Signaling, Regulation of Immune System, Macrophage Activation, and T-Cell Activation. In some samples, some GO processes were not significantly enriched and are not shown.

Figure S14: Histological annotation of BC samples (Reference tissue) and estimation of highly proliferative regions (S+G2M), actively cycling cancer cells and two immune populations (macrophages and $CD8^+$ T cells) in the remaining eight BC samples.

Figure S15: Correlation analysis for all cancer cell populations in the BC atlas, highly proliferative regions (S+G2M) and two immune populations (macrophages and $CD8^+$ T cells).

Figure S16: Gene ontology analysis of the top 5 significantly enriched biological processes in the indicated CAF subpopulations. No significant enrichment was found for Acto-myCAFs.

Figure S17: Box plots showing the patient-wise statistical analysis of *EMILIN1* expression in $CD8^+$ cells/TGFβ+ regions in the indicated ten BC samples.

Figure S18: (**A**) Brightfield image showing the histological structure and cell nuclei of the IF staining from the **Figure 5A**. (**B**) Low- and high-power views of the multiplexed immunofluorescence analysis displaying the localization of EMILIN1, Ki-67 and CD8 in representative BC samples (N=7). Histological structure and cell nuclei are shown using brightfield imaging.

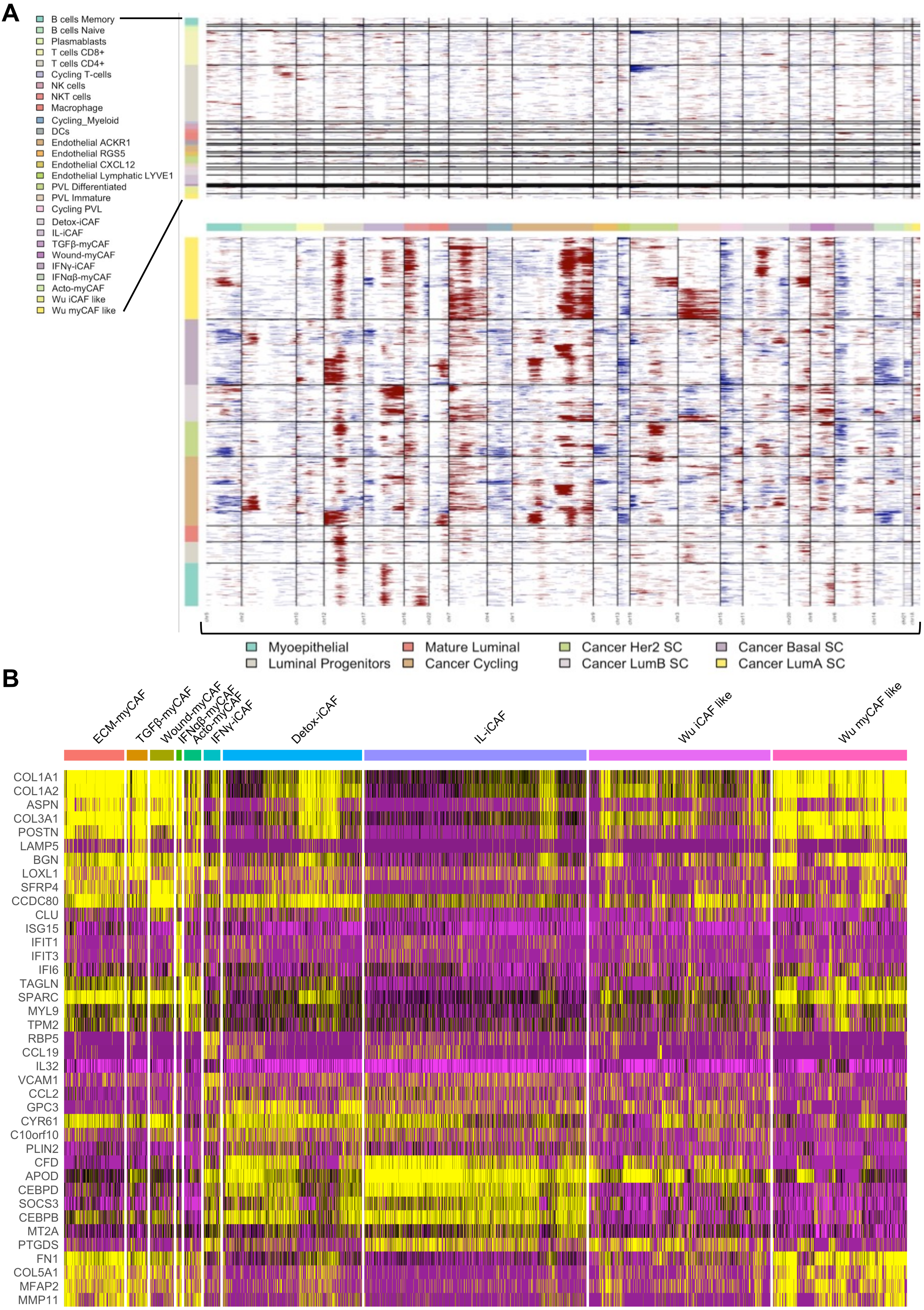
A
B cells Memory
B cells Naive
Plasmablasts
T cells CD8+
T cells CD4+
Cycling T-cells
NK cells
NKT cells
Macrophage
Cycling_Myeloid
DCs
Endothelial ACKR1
Endothelial RGS5
Endothelial CXCL12
Endothelial Lymphatic LYVE1
PVL Differentiated
PVL Immature
Cycling PVL
Detox-iCAF
IL-iCAF
TGFβ-myCAF
Wound-myCAF
IFNγ-iCAF
IFNαβ-myCAF
Acto-myCAF
Wu iCAF like
Wu myCAF like
Myoepithelial
Luminal Progenitors
Mature Luminal
Cancer Cycling
Cancer Her2 SC
Cancer LumB SC
Cancer Basal SC
Cancer LumA SC
B
ECM-myCAF
TGFβ-myCAF
Wound-myCAF
IFNαβ-myCAF
Acto-myCAF
IFNγ-iCAF
Detox-iCAF
IL-iCAF
Wu iCAF like
Wu myCAF like
COL1A1
COL1A2
ASPN
COL3A1
POSTN
LAMP5
BGN
LOXL1
SFRP4
CCDC80
CLU
ISG15
IFIT1
IFIT3
IFI6
TAGLN
SPARC
MYL9
TPM2
RBP5
CCL19
IL32
VCAM1
CCL2
GPC3
CYR61
C10orf10
PLIN2
CFD
APOD
CEBPD
SOCS3
CEBPB
MT2A
PTGDS
FN1
COL5A1
MFAP2
MMP11

Figure S1

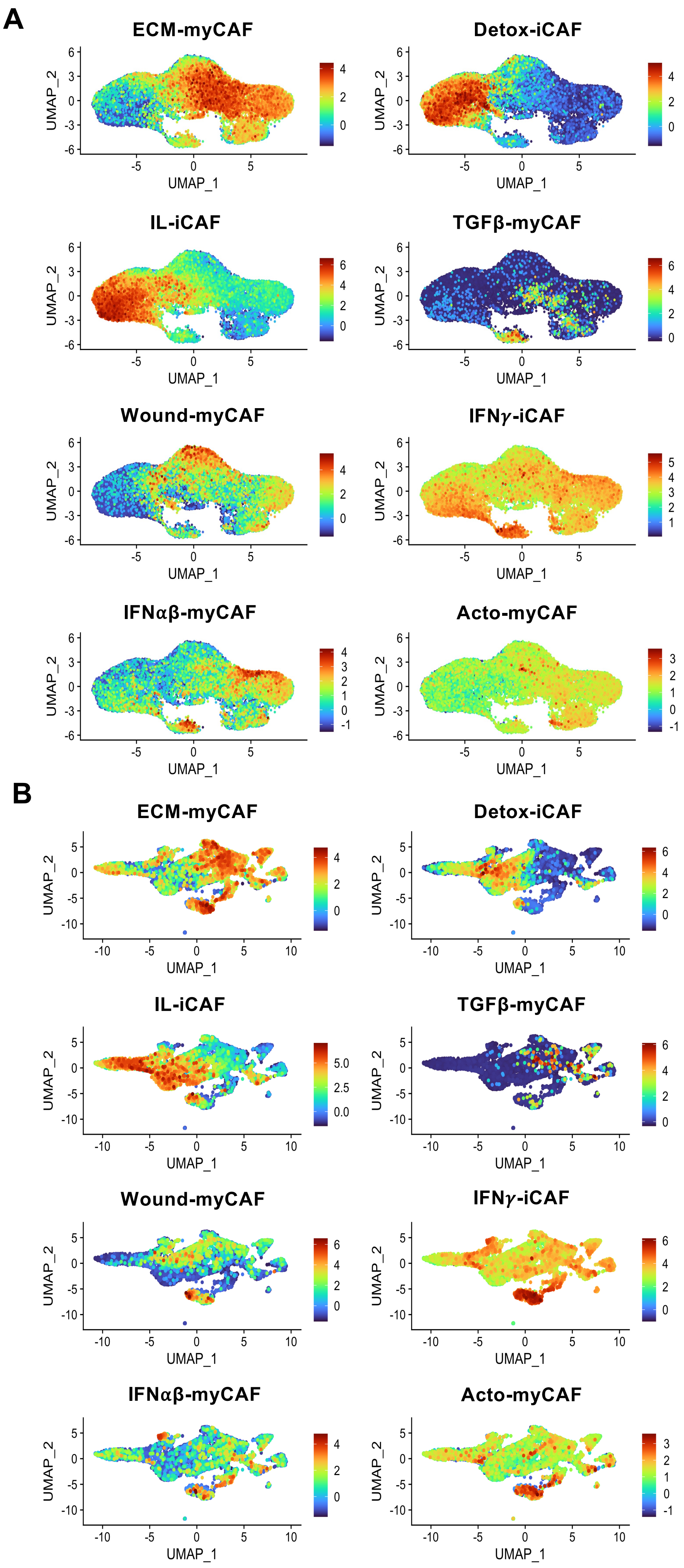
A
ECM-myCAF
Detox-iCAF
IL-iCAF
TGFβ-myCAF
Wound-myCAF
IFNγ-iCAF
IFNαβ-myCAF
Acto-myCAF
UMAP_1
UMAP_2
B
ECM-myCAF
Detox-iCAF
IL-iCAF
TGFβ-myCAF
Wound-myCAF
IFNγ-iCAF
IFNαβ-myCAF
Acto-myCAF
UMAP_1
UMAP_2

Figure S2

A1

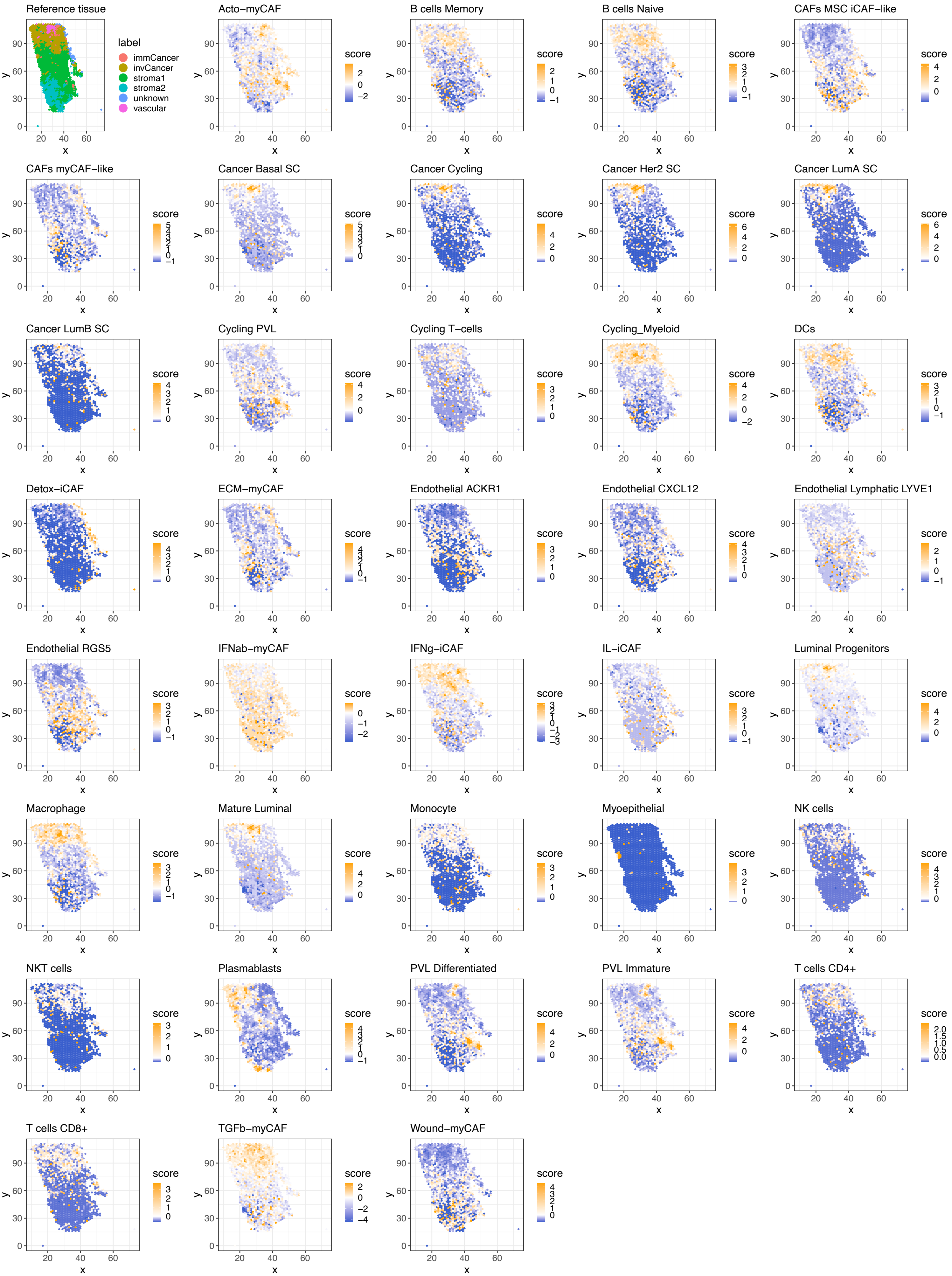
Reference tissue
label
immCancer
invCancer
stroma1
stroma2
unknown
vascular
Acto−myCAF
B cells Memory
B cells Naive
CAFs MSC iCAF−like
CAFs myCAF−like
Cancer Basal SC
Cancer Cycling
Cancer Her2 SC
Cancer LumA SC
Cancer LumB SC
Cycling PVL
Cycling T−cells
Cycling_Myeloid
DCs
Detox−iCAF
ECM−myCAF
Endothelial ACKR1
Endothelial CXCL12
Endothelial Lymphatic LYVE1
Endothelial RGS5
IFNab−myCAF
IFNg−iCAF
IL−iCAF
Luminal Progenitors
Macrophage
Mature Luminal
Monocyte
Myoepithelial
NK cells
NKT cells
Plasmablasts
PVL Differentiated
PVL Immature
T cells CD4+
T cells CD8+
TGFb−myCAF
Wound−myCAF
score
x
y

Figure S3

B1

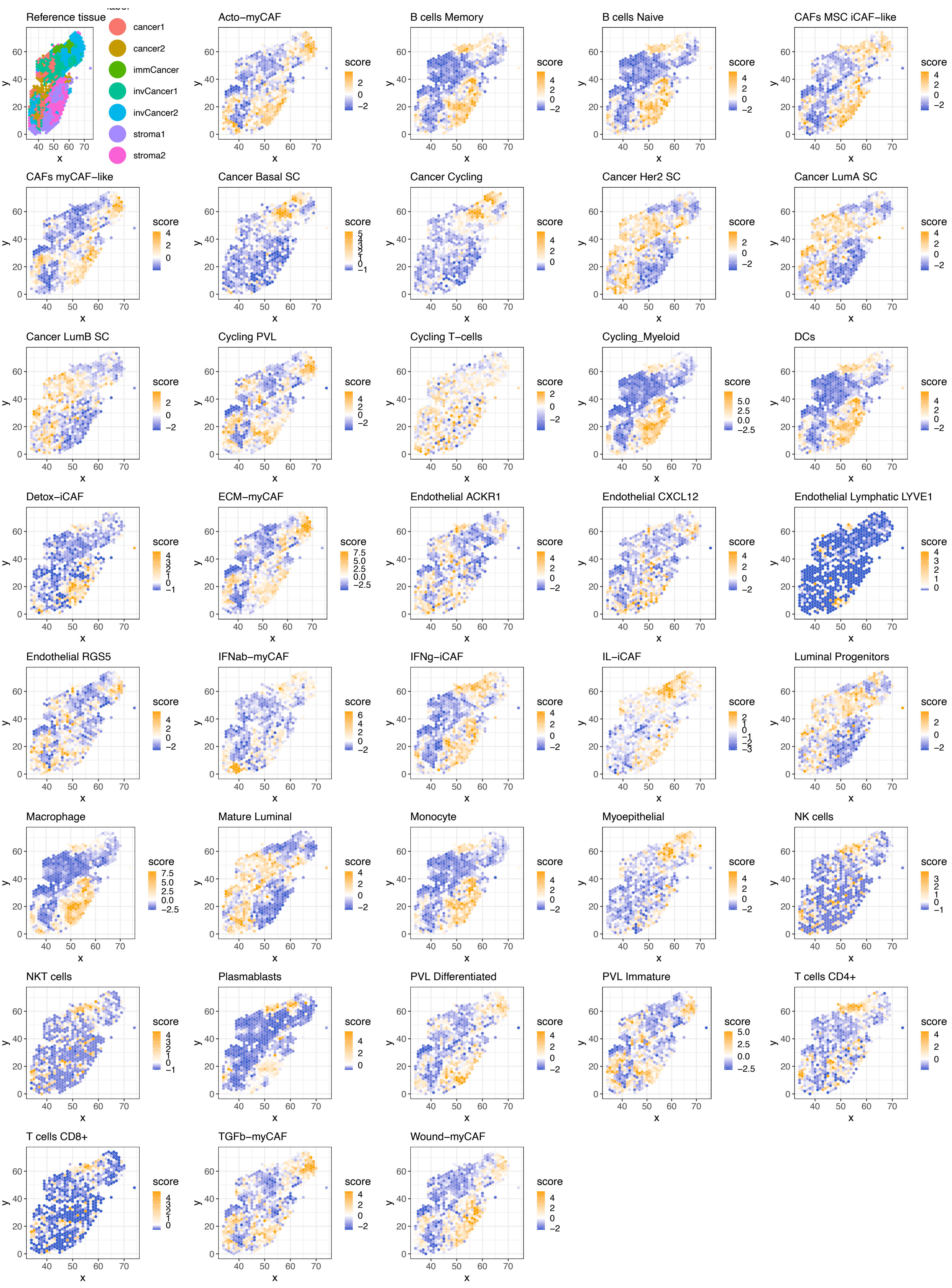

Reference tissue
label
cancer1
cancer2
immCancer
invCancer1
invCancer2
stroma1
stroma2
Acto−myCAF
B cells Memory
B cells Naive
CAFs MSC iCAF−like
CAFs myCAF−like
Cancer Basal SC
Cancer Cycling
Cancer Her2 SC
Cancer LumA SC
Cancer LumB SC
Cycling PVL
Cycling T−cells
Cycling_Myeloid
DCs
Detox−iCAF
ECM−myCAF
Endothelial ACKR1
Endothelial CXCL12
Endothelial Lymphatic LYVE1
Endothelial RGS5
IFNab−myCAF
IFNg−iCAF
IL−iCAF
Luminal Progenitors
Macrophage
Mature Luminal
Monocyte
Myoepithelial
NK cells
NKT cells
Plasmablasts
PVL Differentiated
PVL Immature
T cells CD4+
T cells CD8+
TGFb−myCAF
Wound−myCAF
score
x
y

Figure S4

C1

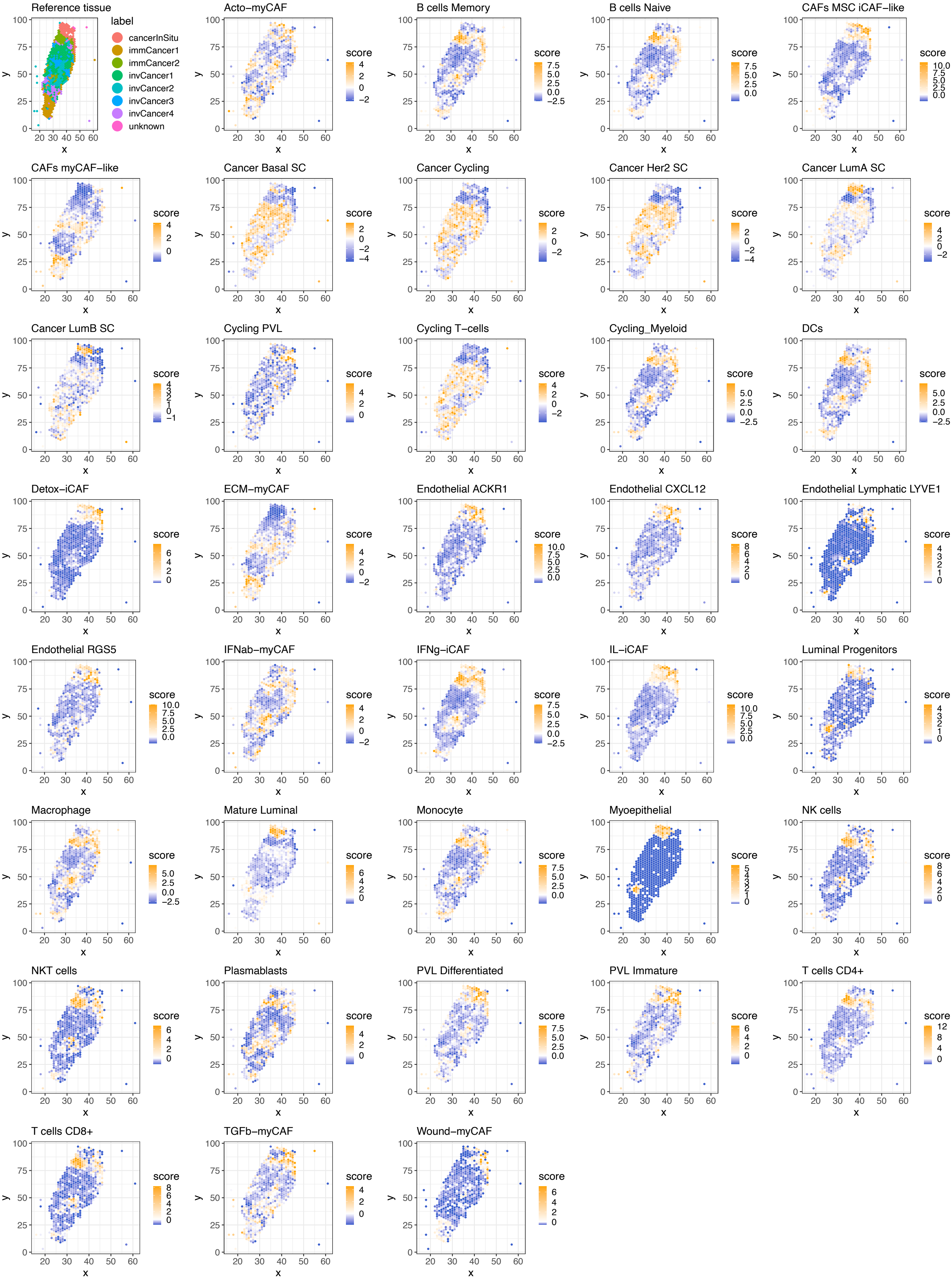

Reference tissue
label
cancerInSitu
immCancer1
immCancer2
invCancer1
invCancer2
invCancer3
invCancer4
unknown
Acto−myCAF
B cells Memory
B cells Naive
CAFs MSC iCAF−like
CAFs myCAF−like
Cancer Basal SC
Cancer Cycling
Cancer Her2 SC
Cancer LumA SC
Cancer LumB SC
Cycling PVL
Cycling T−cells
Cycling_Myeloid
DCs
Detox−iCAF
ECM−myCAF
Endothelial ACKR1
Endothelial CXCL12
Endothelial Lymphatic LYVE1
Endothelial RGS5
IFNab−myCAF
IFNg−iCAF
IL−iCAF
Luminal Progenitors
Macrophage
Mature Luminal
Monocyte
Myoepithelial
NK cells
NKT cells
Plasmablasts
PVL Differentiated
PVL Immature
T cells CD4+
T cells CD8+
TGFb−myCAF
Wound−myCAF
score
x
y

Figure S5

D1

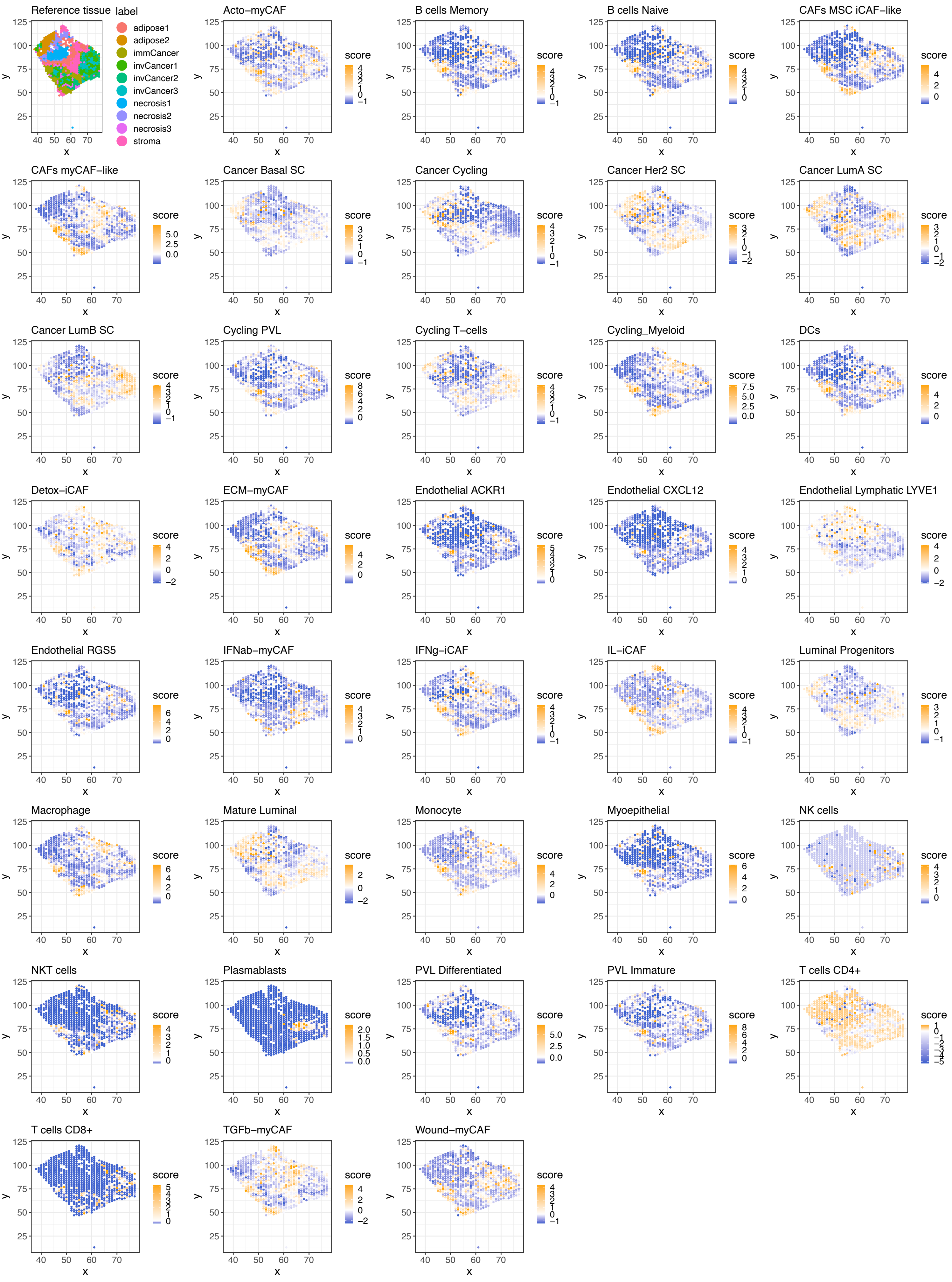

Reference tissue
label
adipose1
adipose2
immCancer
invCancer1
invCancer2
invCancer3
necrosis1
necrosis2
necrosis3
stroma
Acto−myCAF
B cells Memory
B cells Naive
CAFs MSC iCAF−like
CAFs myCAF−like
Cancer Basal SC
Cancer Cycling
Cancer Her2 SC
Cancer LumA SC
Cancer LumB SC
Cycling PVL
Cycling T−cells
Cycling_Myeloid
DCs
Detox−iCAF
ECM−myCAF
Endothelial ACKR1
Endothelial CXCL12
Endothelial Lymphatic LYVE1
Endothelial RGS5
IFNab−myCAF
IFNg−iCAF
IL−iCAF
Luminal Progenitors
Macrophage
Mature Luminal
Monocyte
Myoepithelial
NK cells
NKT cells
Plasmablasts
PVL Differentiated
PVL Immature
T cells CD4+
T cells CD8+
TGFb−myCAF
Wound−myCAF
score
x
y

Figure S6

1160920F

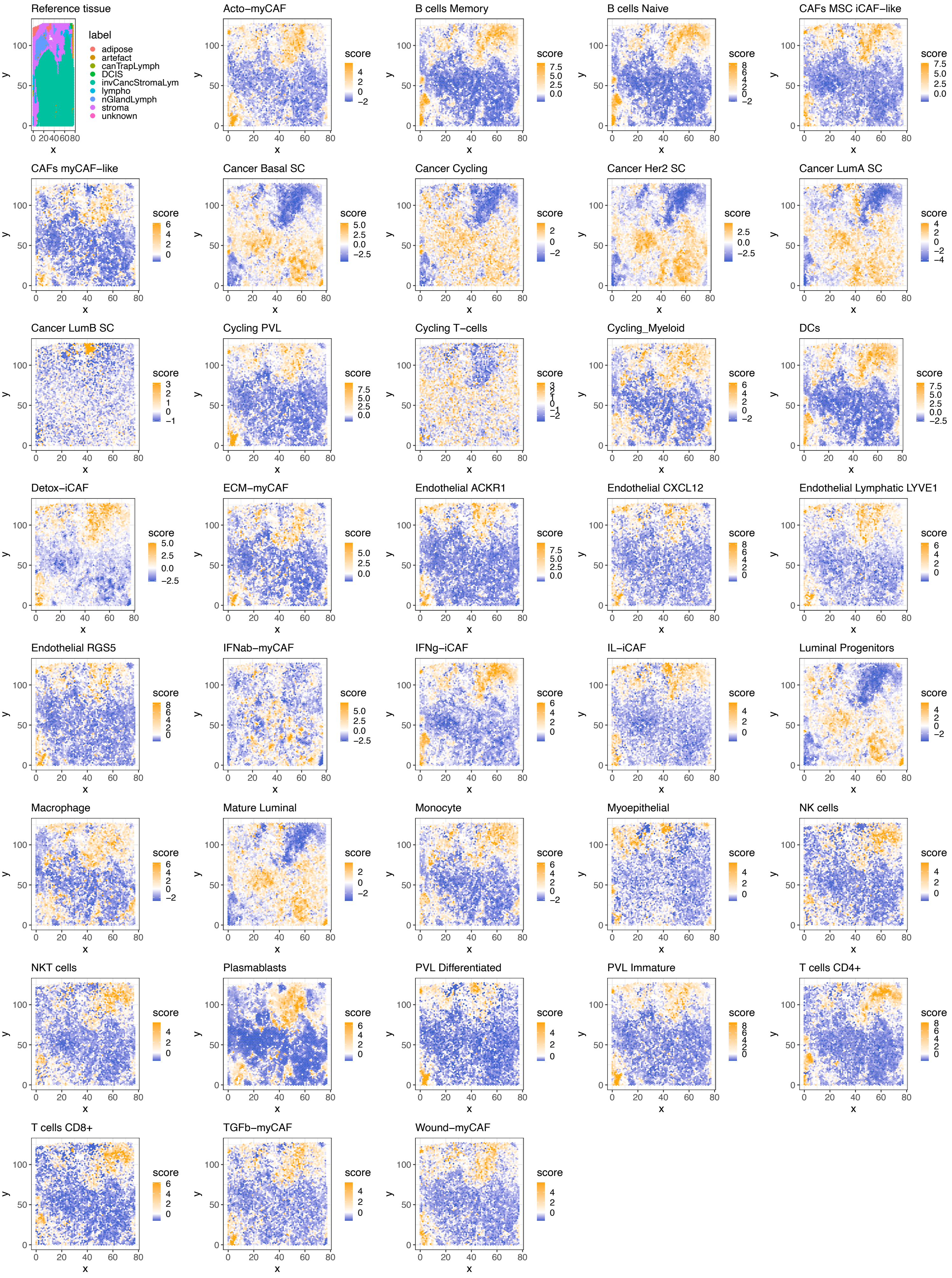

Figure S7

1124423F

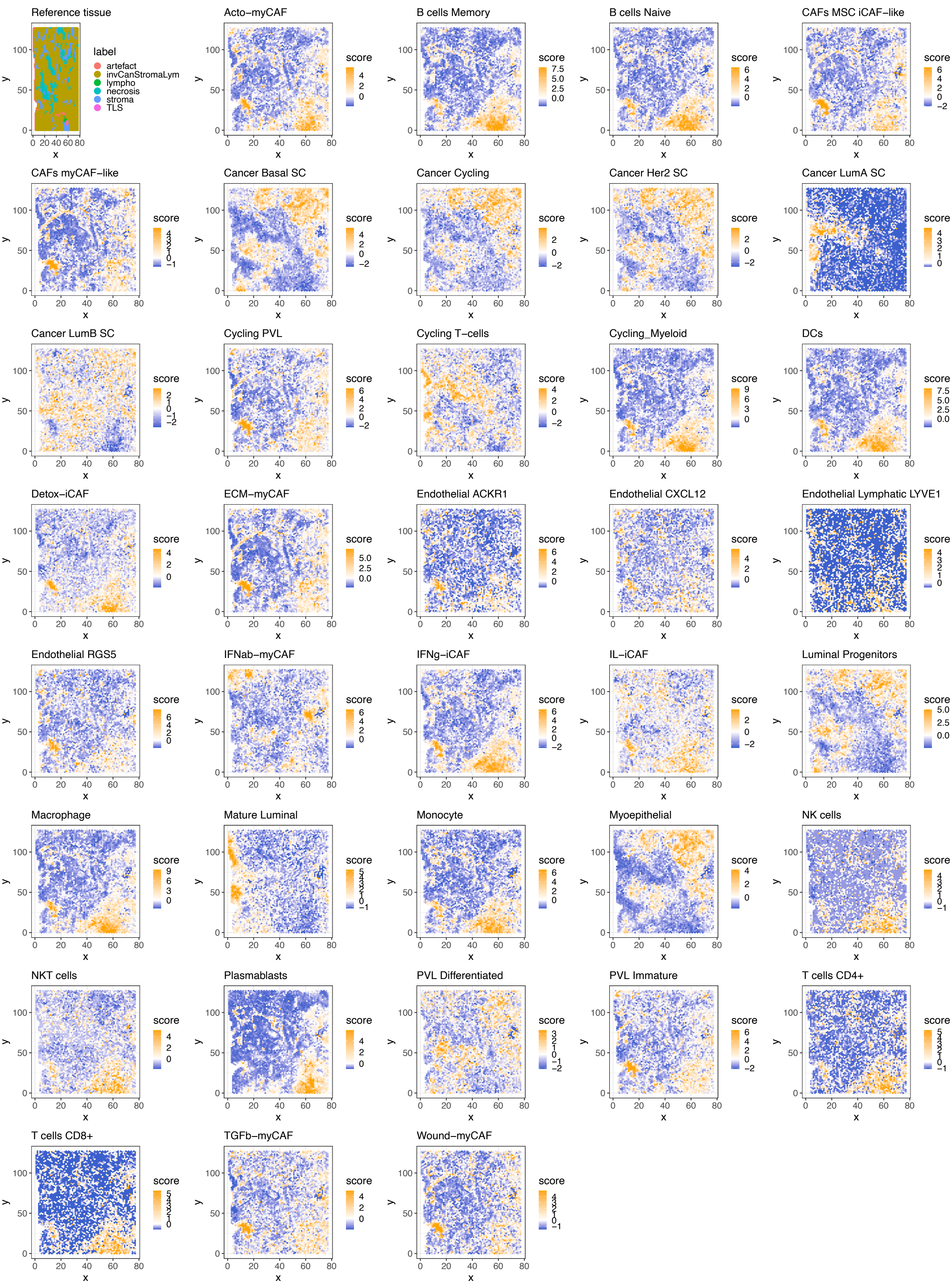

Figure S8

CID4290

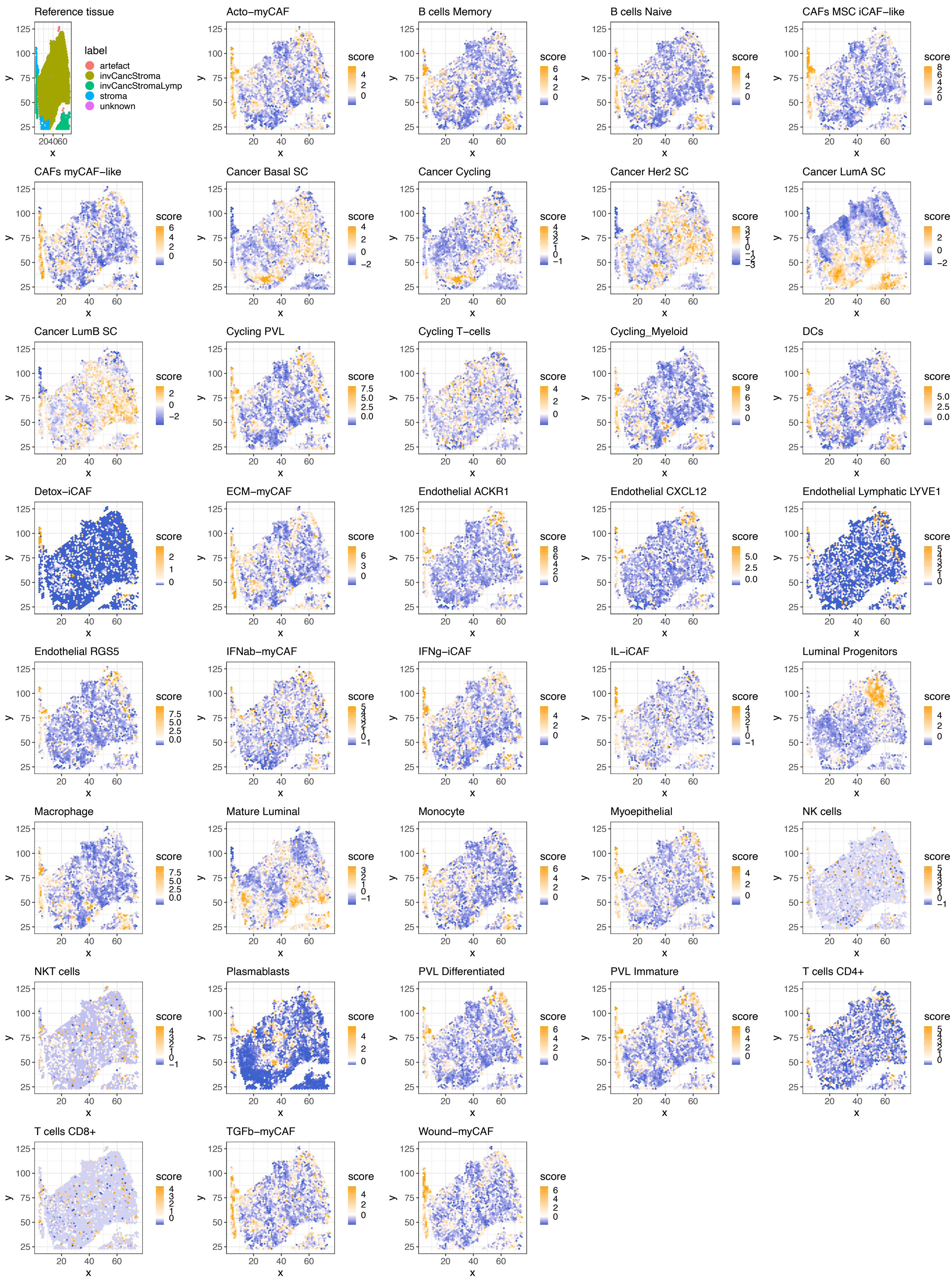

Figure S9

CID44971

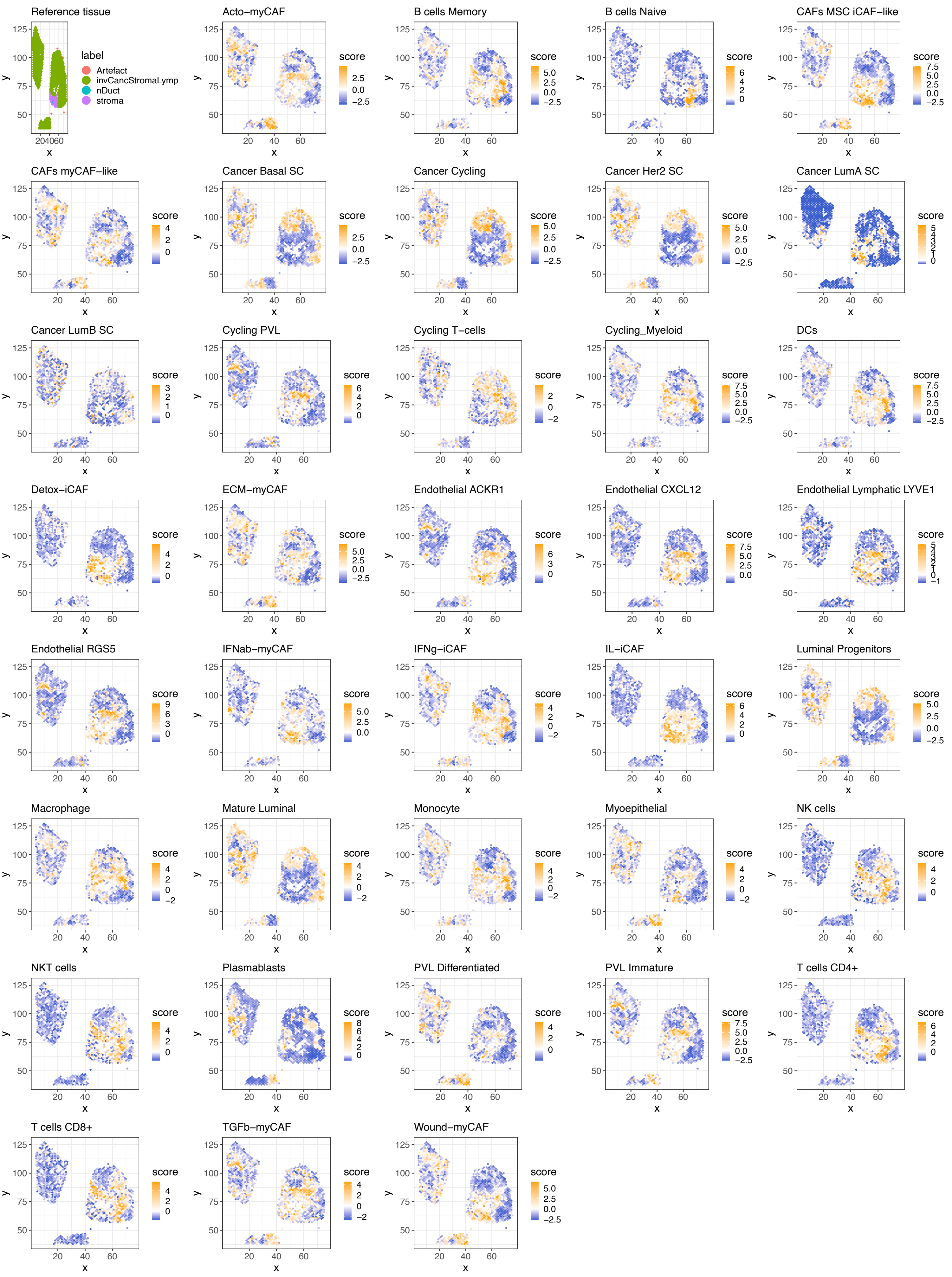

Figure S10

CID4465

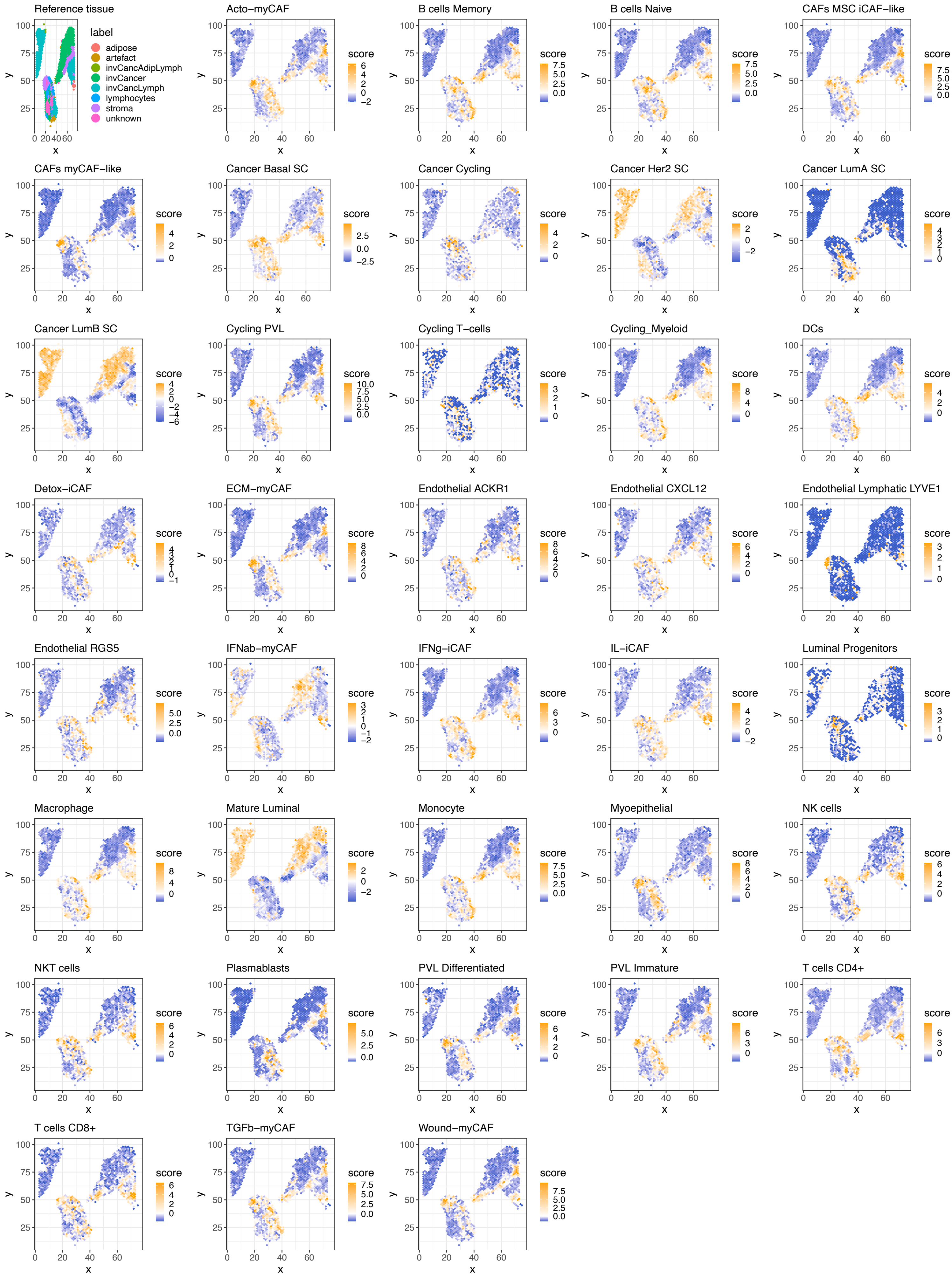

Figure S11

CID4535

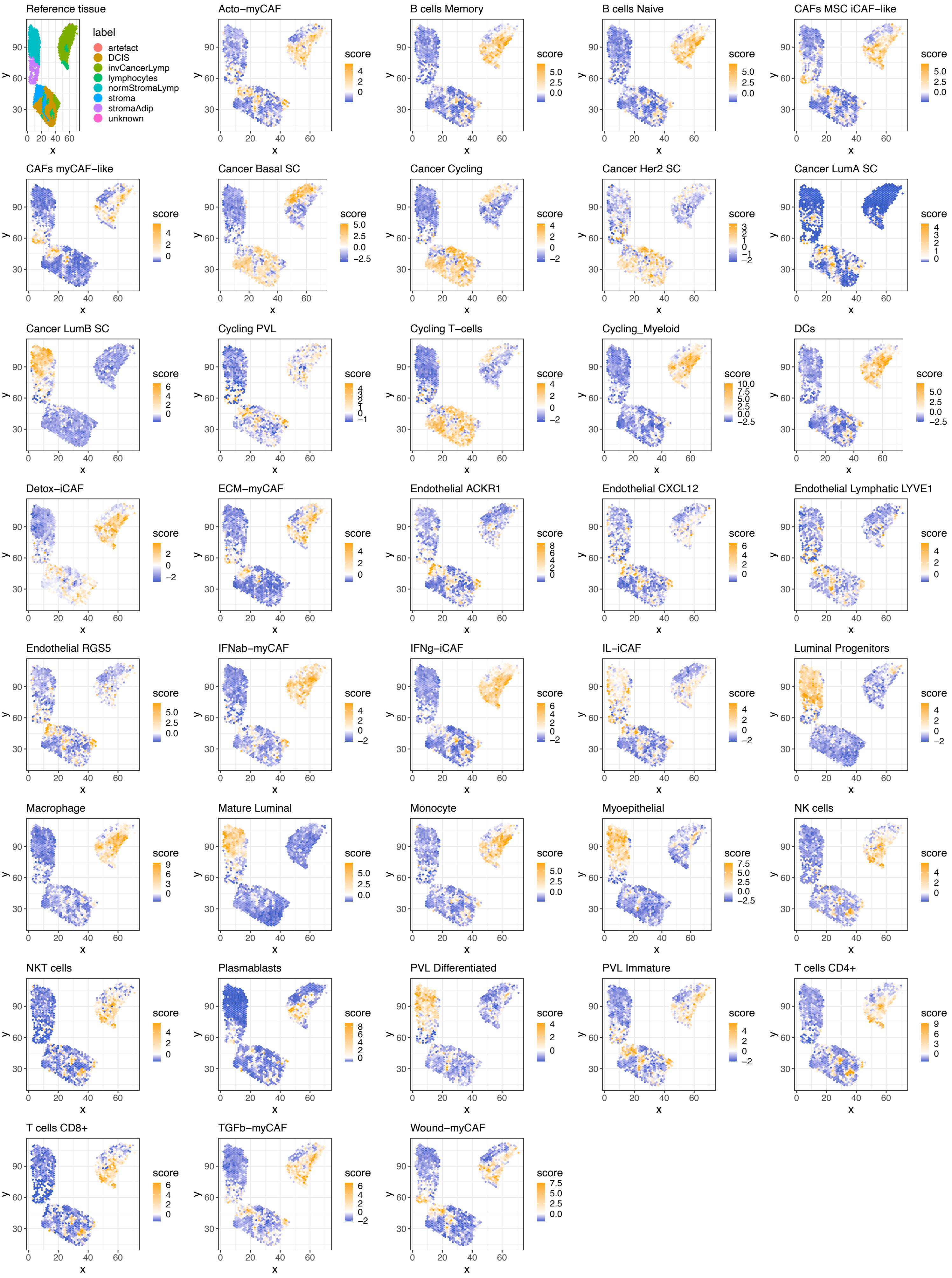

Figure S12

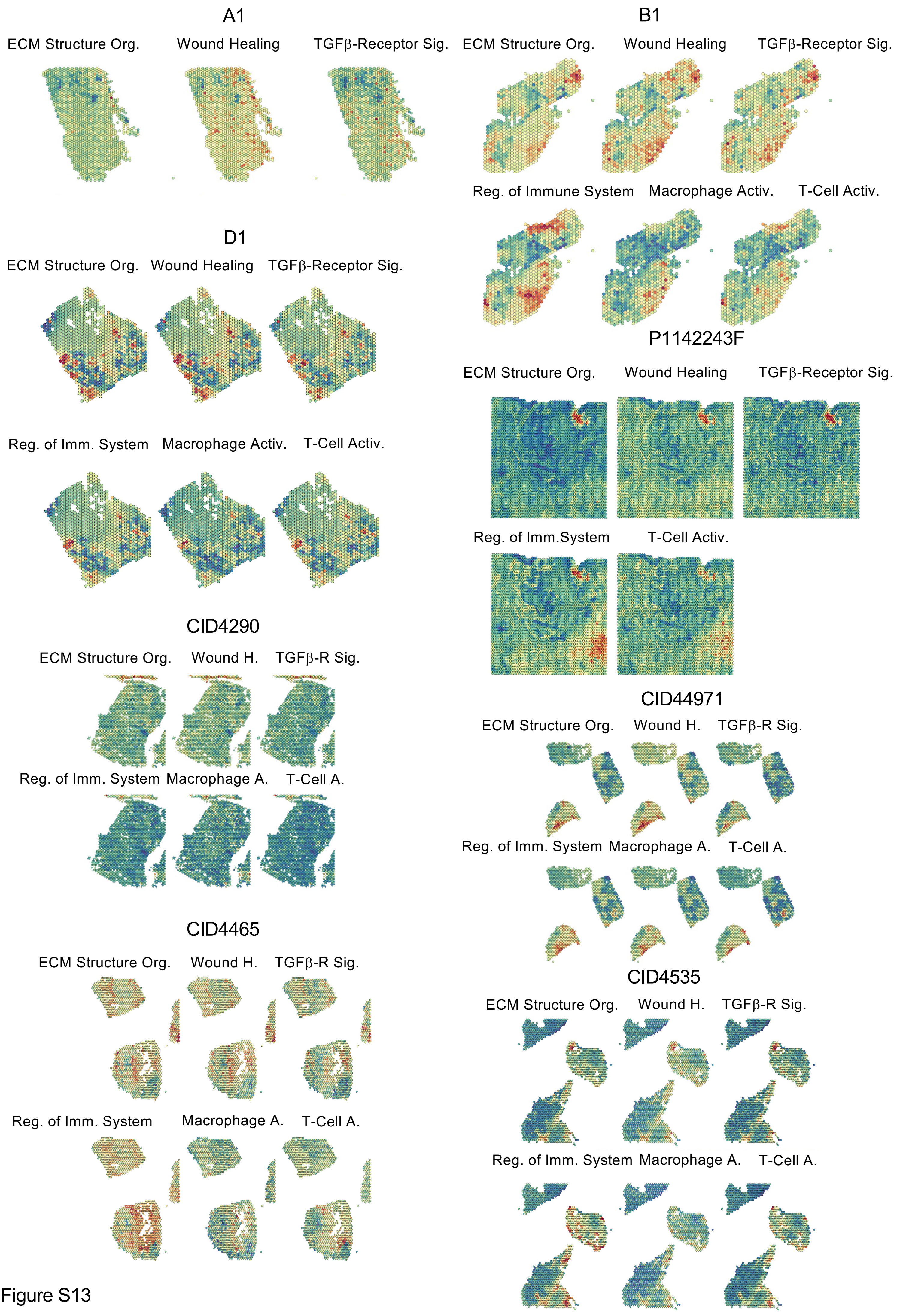
A1
ECM Structure Org.
Wound Healing
TGFβ-Receptor Sig.
B1
ECM Structure Org.
Wound Healing
TGFβ-Receptor Sig.
Reg. of Immune System
Macrophage Activ.
T-Cell Activ.
D1
ECM Structure Org.
Wound Healing
TGFβ-Receptor Sig.
Reg. of Imm. System
Macrophage Activ.
T-Cell Activ.
P1142243F
ECM Structure Org.
Wound Healing
TGFβ-Receptor Sig.
Reg. of Imm.System
T-Cell Activ.
CID4290
ECM Structure Org.
Wound H.
TGFβ-R Sig.
Reg. of Imm. System
Macrophage A.
T-Cell A.
CID44971
ECM Structure Org.
Wound H.
TGFβ-R Sig.
Reg. of Imm. System
Macrophage A.
T-Cell A.
CID4465
ECM Structure Org.
Wound H.
TGFβ-R Sig.
Reg. of Imm. System
Macrophage A.
T-Cell A.
CID4535
ECM Structure Org.
Wound H.
TGFβ-R Sig.
Reg. of Imm. System
Macrophage A.
T-Cell A.

Figure S13

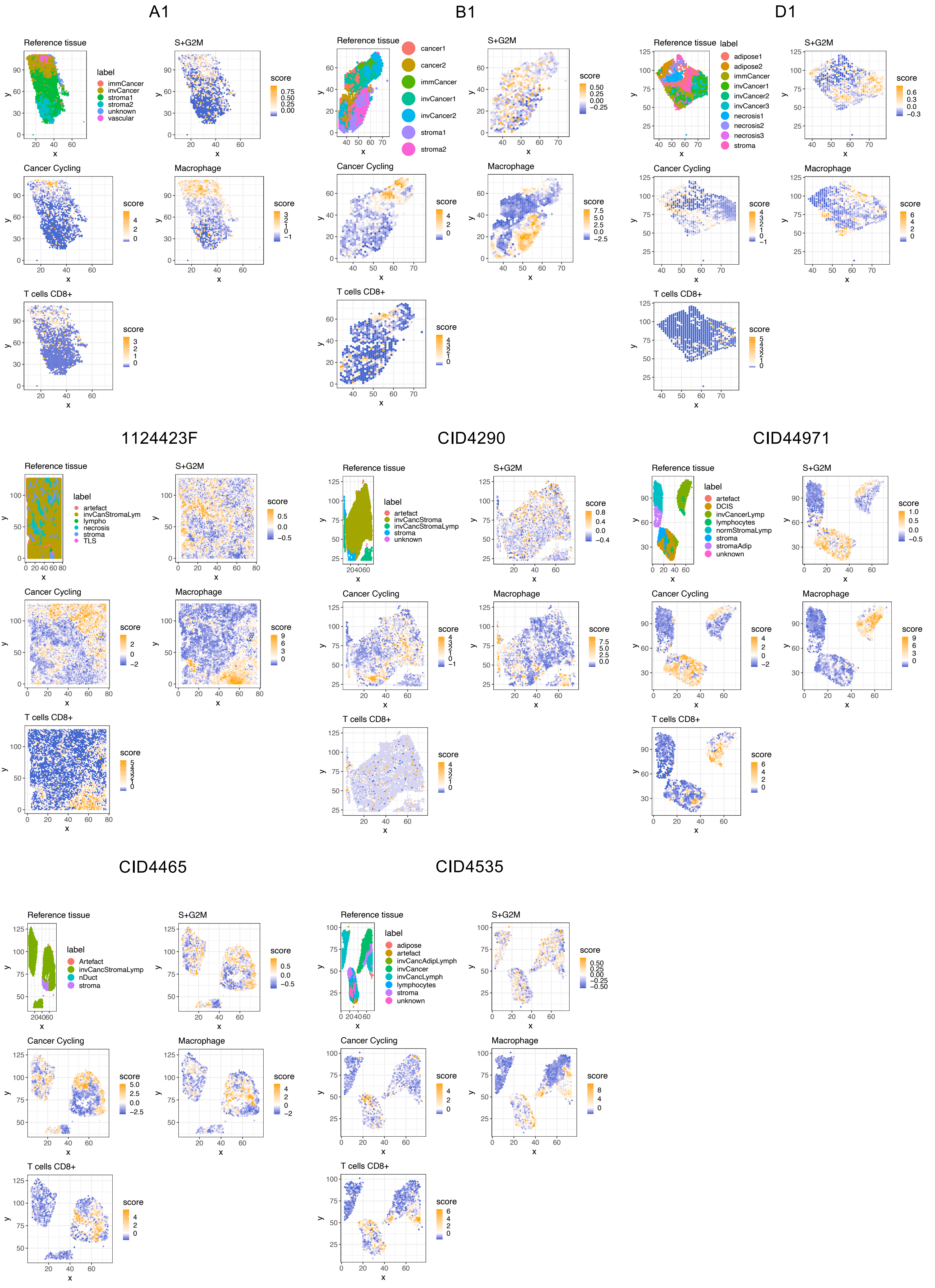
A1
B1
D1
1124423F
CID4290
CID44971
CID4465
CID4535
Reference tissue
S+G2M
Cancer Cycling
Macrophage
T cells CD8+
label
score
x
y
immCancer
invCancer
stroma1
stroma2
unknown
vascular
cancer1
cancer2
invCancer1
invCancer2
adipose1
adipose2
invCancer3
necrosis1
necrosis2
necrosis3
stroma
artefact
invCanStromaLym
lympho
necrosis
TLS
invCancStroma
invCancStromaLymp
DCIS
invCancLymp
lymphocytes
normStromaLymp
stromaAdip
Artefact
nDuct
adipose
invCancAdipLymph
invCancLymph

Figure S14

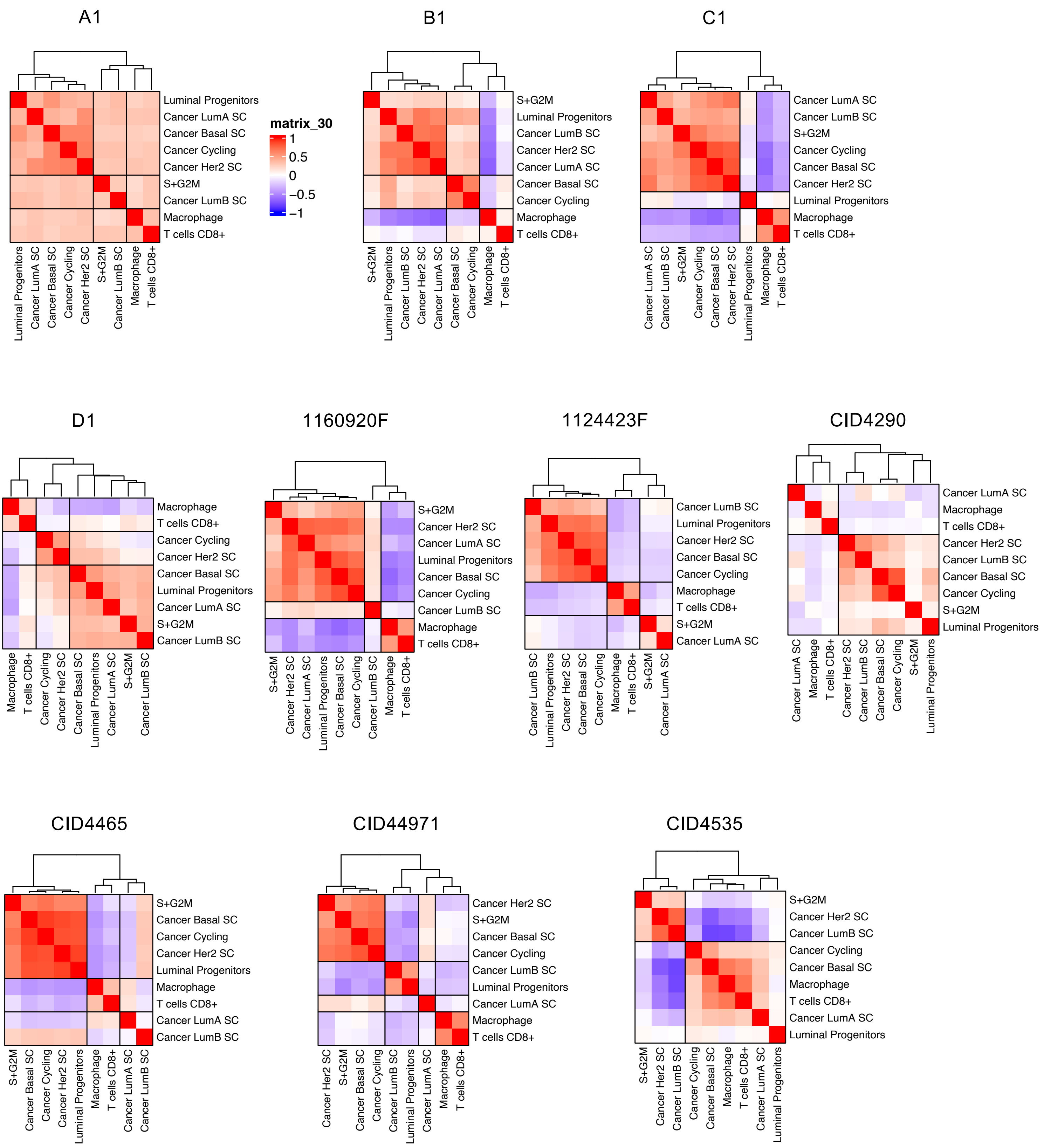
A1
Luminal Progenitors
Cancer LumA SC
Cancer Basal SC
Cancer Cycling
Cancer Her2 SC
S+G2M
Cancer LumB SC
Macrophage
T cells CD8+
matrix_30
1
0.5
0
−0.5
−1
B1
S+G2M
Luminal Progenitors
Cancer LumB SC
Cancer Her2 SC
Cancer LumA SC
Cancer Basal SC
Cancer Cycling
Macrophage
T cells CD8+
C1
Cancer LumA SC
Cancer LumB SC
S+G2M
Cancer Cycling
Cancer Basal SC
Cancer Her2 SC
Luminal Progenitors
Macrophage
T cells CD8+
D1
Macrophage
T cells CD8+
Cancer Cycling
Cancer Her2 SC
Cancer Basal SC
Luminal Progenitors
Cancer LumA SC
S+G2M
Cancer LumB SC
1160920F
S+G2M
Cancer Her2 SC
Cancer LumA SC
Luminal Progenitors
Cancer Basal SC
Cancer Cycling
Cancer LumB SC
Macrophage
T cells CD8+
1124423F
Cancer LumB SC
Luminal Progenitors
Cancer Her2 SC
Cancer Basal SC
Cancer Cycling
Macrophage
T cells CD8+
S+G2M
Cancer LumA SC
CID4290
Cancer LumA SC
Macrophage
T cells CD8+
Cancer Her2 SC
Cancer LumB SC
Cancer Basal SC
Cancer Cycling
S+G2M
Luminal Progenitors
CID4465
S+G2M
Cancer Basal SC
Cancer Cycling
Cancer Her2 SC
Luminal Progenitors
Macrophage
T cells CD8+
Cancer LumA SC
Cancer LumB SC
CID44971
Cancer Her2 SC
S+G2M
Cancer Basal SC
Cancer Cycling
Cancer LumB SC
Luminal Progenitors
Cancer LumA SC
Macrophage
T cells CD8+
CID4535
S+G2M
Cancer Her2 SC
Cancer LumB SC
Cancer Cycling
Cancer Basal SC
Macrophage
T cells CD8+
Cancer LumA SC
Luminal Progenitors

Figure S15

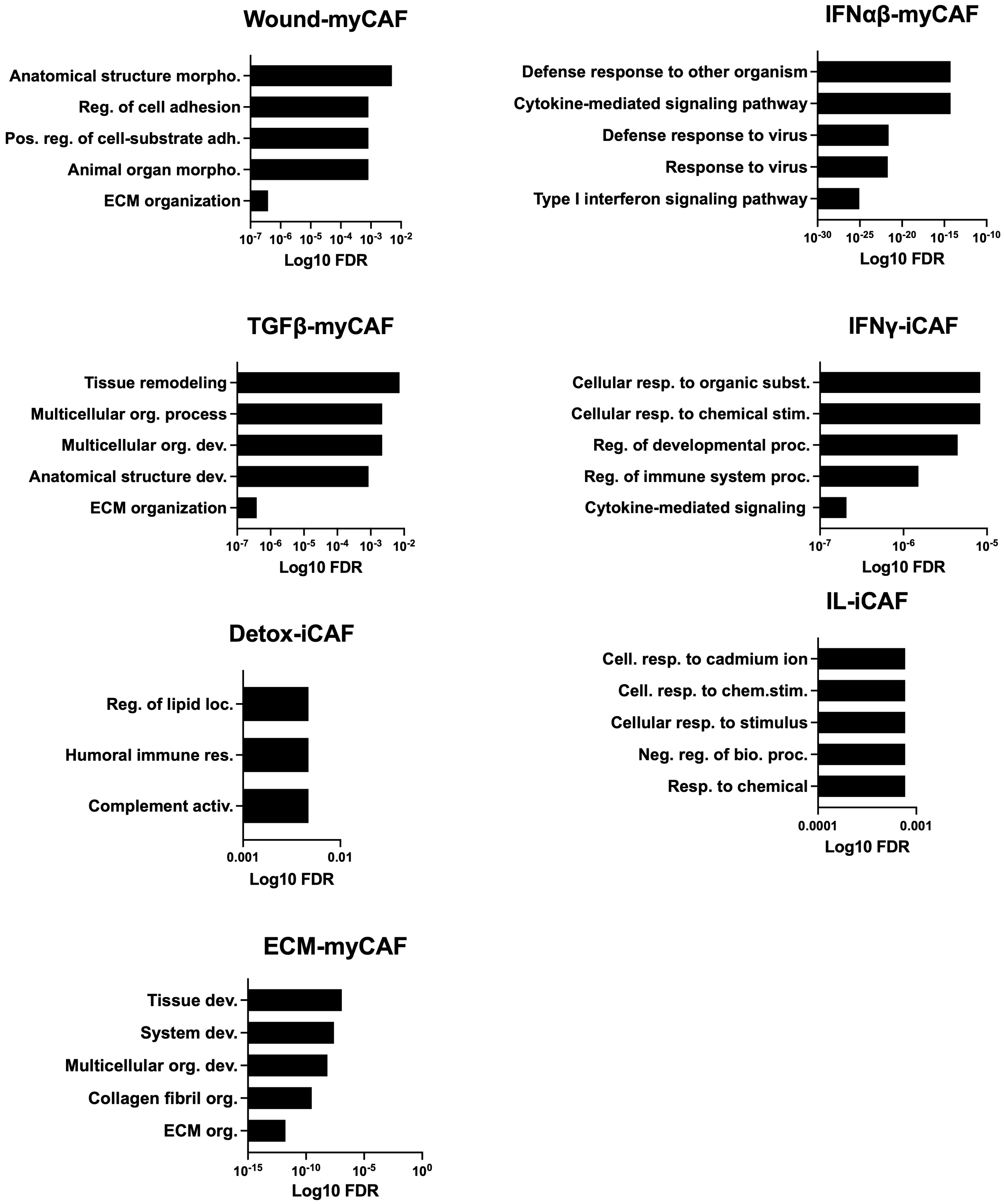

Wound-myCAF
Anatomical structure morpho.
Reg. of cell adhesion
Pos. reg. of cell-substrate adh.
Animal organ morpho.
ECM organization
10^-7 10^-6 10^-5 10^-4 10^-3 10^-2
Log10 FDR
IFNαβ-myCAF
Defense response to other organism
Cytokine-mediated signaling pathway
Defense response to virus
Response to virus
Type I interferon signaling pathway
10^-30 10^-25 10^-20 10^-15 10^-10
Log10 FDR
TGFβ-myCAF
Tissue remodeling
Multicellular org. process
Multicellular org. dev.
Anatomical structure dev.
ECM organization
10^-7 10^-6 10^-5 10^-4 10^-3 10^-2
Log10 FDR
IFNγ-iCAF
Cellular resp. to organic subst.
Cellular resp. to chemical stim.
Reg. of developmental proc.
Reg. of immune system proc.
Cytokine-mediated signaling
10^-7 10^-6 10^-5
Log10 FDR
IL-iCAF
Cell. resp. to cadmium ion
Cell. resp. to chem.stim.
Cellular resp. to stimulus
Neg. reg. of bio. proc.
Resp. to chemical
0.0001 0.001
Log10 FDR
Detox-iCAF
Reg. of lipid loc.
Humoral immune res.
Complement activ.
0.001 0.01
Log10 FDR
ECM-myCAF
Tissue dev.
System dev.
Multicellular org. dev.
Collagen fibril org.
ECM org.
10^-15 10^-10 10^-5 10^0
Log10 FDR

Figure S16

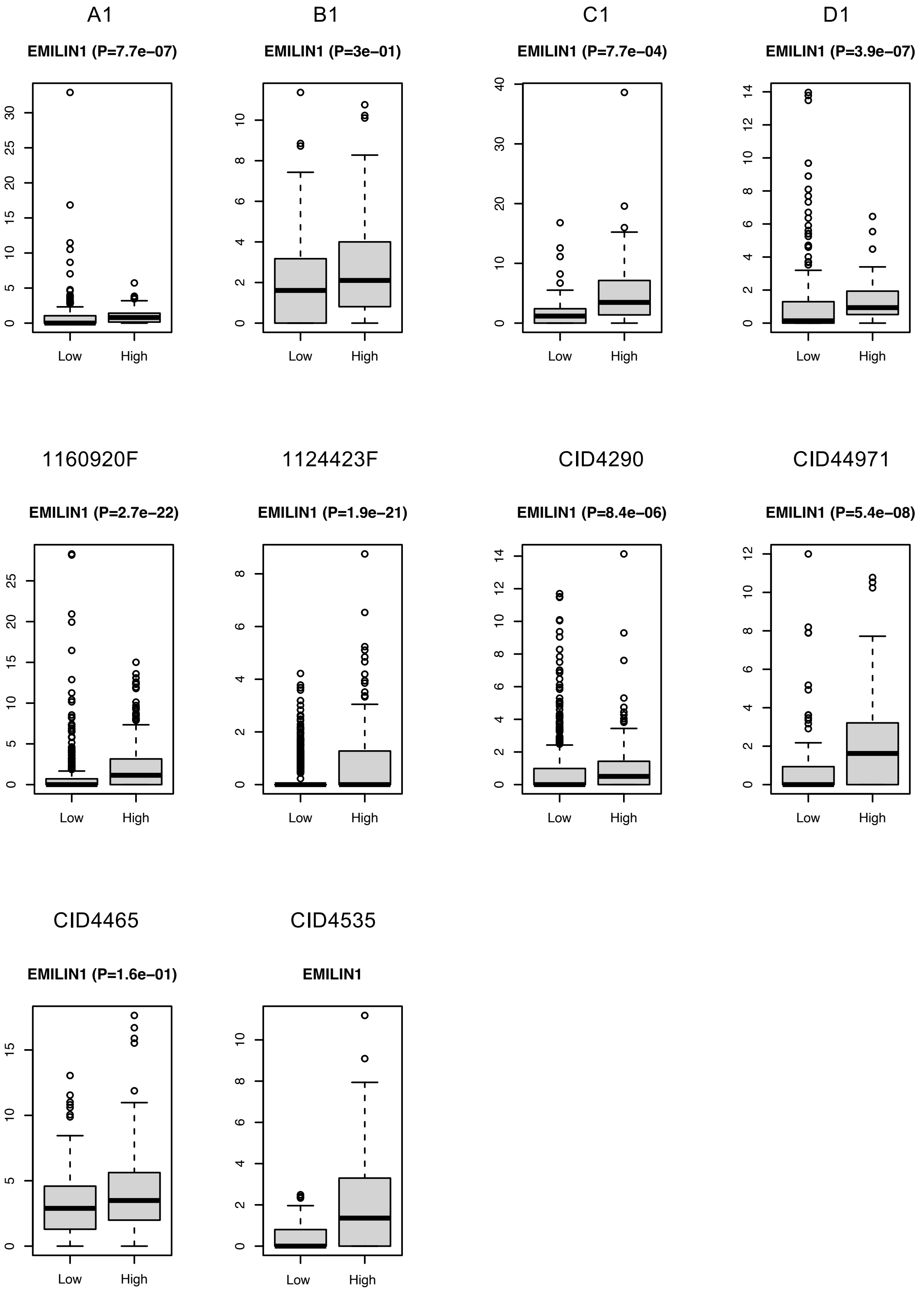
A1
EMILIN1 (P=7.7e−07)
B1
EMILIN1 (P=3e−01)
C1
EMILIN1 (P=7.7e−04)
D1
EMILIN1 (P=3.9e−07)
1160920F
EMILIN1 (P=2.7e−22)
1124423F
EMILIN1 (P=1.9e−21)
CID4290
EMILIN1 (P=8.4e−06)
CID44971
EMILIN1 (P=5.4e−08)
CID4465
EMILIN1 (P=1.6e−01)
CID4535
EMILIN1
Low
High

Figure S17

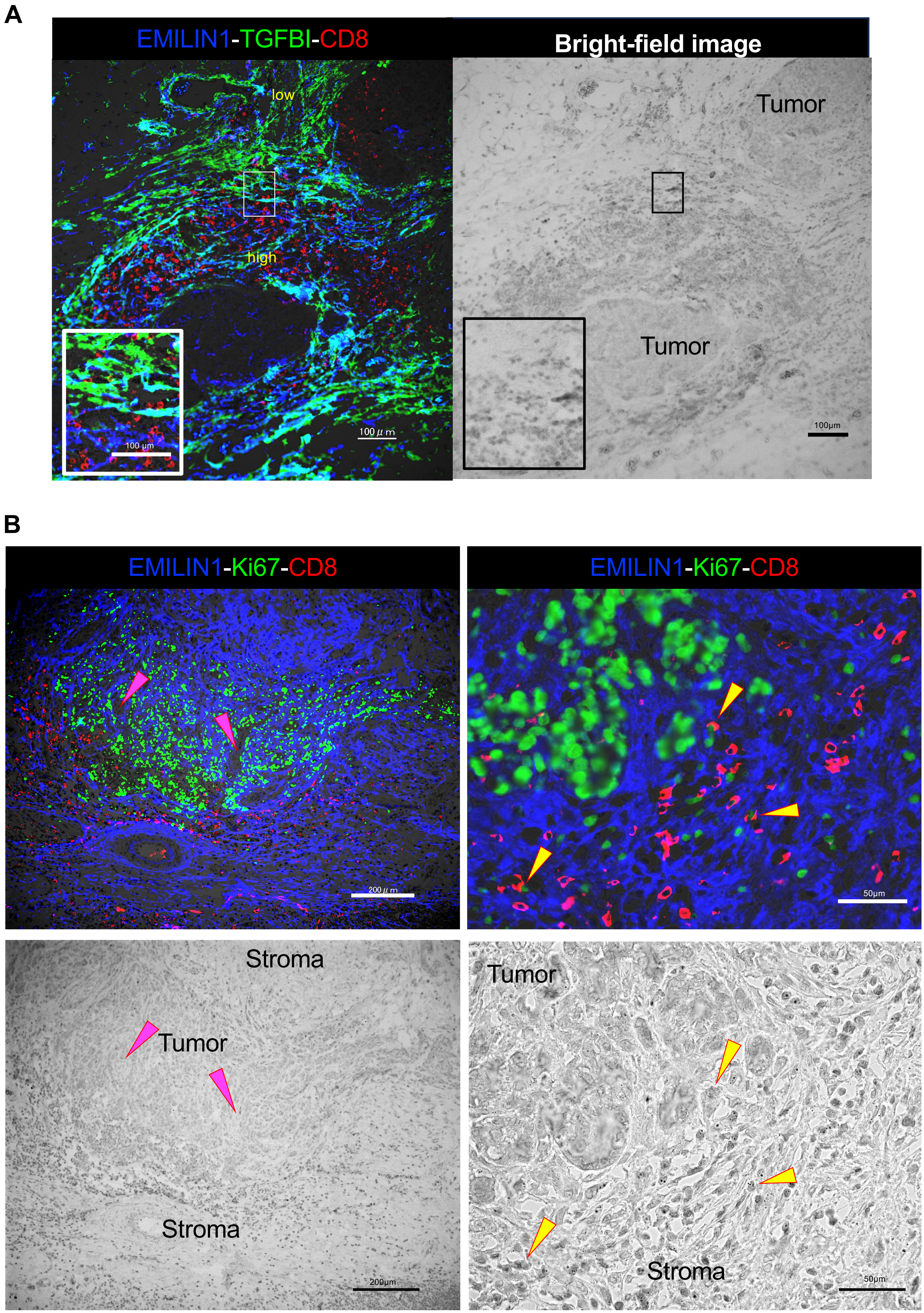
A
EMILIN1-TGFBI-CD8
low
high
100 μm
100 μm
Bright-field image
Tumor
Tumor
100μm
B
EMILIN1-Ki67-CD8
200 μm
EMILIN1-Ki67-CD8
50μm
Stroma
Tumor
Stroma
200μm
Tumor
Stroma
50μm

Figure S18

Table S1: Clinicopathological factors of 4 spatial transcriptomic cases

Patient	A1	B1	C1	D1
Age	89	79	52	56
Histological subtype	IDC	IDC	IDC	IDC
ER	1%＞	1%＞	1%≤	1%≤
PgR	10%＞	10%＞	10%≦	10%≦
HER2	3+	1+	3+	2+ (FISH negative)
T factor	T2	T2	T1c	T3
N factor	3a	0	0	0
M factor	0	0	0	0
SBR Grade	3	3	3	3
Stage (UICC 8th)	IIIC	IIA	I	IIB
Ki-67（%）	12−17	7--15	unknown	27-32
Lymphatic invasion	1+	0	1+	1+
Vascular invasion	1+	0	1+	0

Table S2: Clinical features of 75 case strong validation cohort

Factors	Number
Age	
<60	34
60≤	41
Histologic subtype	
IDC	59
ILC	6
Others	10
T factor	
T1	38
T2-4	37
N factor	
N0	47
N1-3	28
M factor	
0	75
1	0
Nuclear Grade	
1	11
2	10
3	54
Stage	
I	34
II-III	41
ER	
1%≦	39
1%＞	36
PgR	
10%≦	25
10%＞	50
HER2	
0	2
1+	34
2+	6
3+	33
Lymphatic invasion	
0	35
1	29
2	10
3	1
Vascular invasion	
0	50
1	24
2	1
3	0
EMILIN	
Low	18
High	57

Table S3: Gene signatures for individual cell subpopulations

Endothelial ACKR1	Endothelial RGS5	Endothelial CXCL12	Endothelial Lymphatic LYVE1	Wu iCAF-like	Wu myCAF-like	ECM-myCAF	Detox-iCAF	IL-iCAF	TGFβ-myCAF
ACKR1	FABP4	IGFBP3	CCL21	APOD	COL1A1	POSTN	CFD	APOD	CST1
PLVAP	PLVAP	FABP4	MMRN1	DCN	COL1A2	FN1	ADH1B	MT2A	LAMP5
VWF	RBP7	CLDN5	PROX1	PTGDS	COL3A1	COL1A1	GPC3	RASD1	HOPX
AQP1	COL4A1	RAMP2	SDPR	CFD	LUM	ACTA2	C10orf10	MT1A	BGN
SPARCL1	HSPG2	SLC9A3R2	CLDN5	LUM	SFRP2	SPARC	GPX3	IRF1	LOXL1
RAMP3	RAMP2	PLPP1	ECSCR.1	C1S	POSTN	ASPN	C3	PDK4	TIMP1
RAMP2	COL4A2	A2M	RAMP2	CXCL12	MMP11	COL1A2	APOC1	MT1M	F2R
ENG	GNG11	CAV1	FABP4	C3	CTHRC1	CTHRC1	EFEMP1	CEBPD	IGFBP7
FABP4	EGFL7	GNG11	GNG11	SFRP2	FN1	COL3A1	SOD2	KLF4	EDNRA
PECAM1	A2M	CLEC14A	EGFL7	CXCL14	SPARC	TAGLN	MGP	SOCS3	NREP
SPRY1	IGFBP7	RBP7	TFPI	CCDC80	DCN	COL5A2	SFRP1	PTGDS	TGFB3
EGFL7	VWF	C10orf10	NRP2	MFAP4	COL6A3	MMP11	LEPR	ZFP36L2	POSTN
IGFBP7	SPARCL1	TM4SF1	AKAP12	FBLN1	BGN	SDC1	C7	JUNB	MMP3
CLDN5	CALCRL	ID1	EFEMP1	GSN	COL6A2	COL5A1	CXCL12	ITM2A	RAMP1
NPDC1	AQP1	EGFL7	TFF3	CTSK	COL6A1	GJB2	IL6	RGMA	MMP11
EMCN	ESAM	CXCL12	PPFIBP1	SERPINF1	CTGF	SERPINH1	PLIN2	GSN	COL10A1
IGFBP4	CD36	ESAM	CAV1	RARRES2	AEBP1	VCAN	CXCL2	ADIRF	MGP
PRCP	PLPP1	PECAM1	RCAN1	C1R	COL5A2	COL10A1		BTG2	ELN
HSPG2	RAMP3	IFI27	FABP5	IGF2	VCAN	THY1		CFD	CXCL14
GNG11	SPARC	ICAM2	CD9	CYR61	CTSK	TPM1		CXCL12	COMP

Wound-myCAF	IFNγ-iCAF	IFNαβ-myCAF	Acto-myCAF	myCAF	iCAF	CAFs1	CAFs4	PVL Differentiated	PVL Immature
SFRP4	B2M	ISG15	ATP5E	COL1A2	SOD2	MMP2	MCAM	ACTA2	CCL19
CCDC80	CCL19	IFI6	PLP2	TAGLN	CXCL12	MFAP5	MEF2C	TAGLN	RGS5
FBLN1	VCAM1	MX1	OST4	BGN	PLA2G2A	LAMA2	MOCS1	MYL9	IGFBP7
OGN	HLA-B	OAS1	SERF2	TPM2	MCL1	SFRP4	PTP4A3	TPM2	NDUFA4L2
PTGER3	CD74	IFI44L	SMYD3	IGFBP7	S100A10	LUM	NRIP2	NDUFA4L2	CCL2
SFRP2	RBP5	RSAD2	MFAP5	MMP11	S100A4	PDGFRA	MYH11	SOD3	CCL21
DHRS3	CLSTN3	IFIT1	DIO2	CST1	ABL2	LRRC15	PARM1	ADIRF	COL18A1
PDGFRL	C3	LY6E	IGFBP7	ACTA2	CXCL1	GREM1	CDH6	MYH11	CALD1
CLU	TYMP	IFIT3	HOPX	POSTN	CXCL2	SFRP2	GJA4	RGS5	LHFP
SMOC2	IL32	IFI27	PPIC	CTHRC1	PTX3	KIAA1462	CACNB2	RERGL	THY1
IGFBP6	PGF	IFITM3	HCFC1R1	CALD1	HAS1	CCL11		IGFBP7	CPE
MFAP5	CTSH	EPSTI1	IGKC	INHBA	FBLN2	CXCL12		CALD1	MYL9
CYR61	PSME2	MX2	CTTN	GRP		CXCL13		SPARCL1	SPARC
CD9	CYP1B1	IFI44	SPARC			CXCL14		MT1M	COL4A1
COL1A2	BIRC3	STAT1	CDKN2A					C11orf96	TAGLN
CTGF	PTGDS	CXCL10	TAGLN					PPP1R14A	STEAP4
WISP2	APOE	IFIT2	TGFB3					MFGE8	ACTA2
COL8A1	CXCL9		PALLD					PLAC9	COL4A2
PRSS23	CCL2		IFI27					DSTN	TIMP1
CITED2	CXCL10		IGHM					PTP4A3	IGFBP5

Cycling PVL	B cells Memory	B cells Naive	Plasmablasts	T cells CD8+	T cells CD4+	NK cells	Cycling T-cells	NKT cells	Macrophage
NDUFA4L2	MS4A1	CD79A	IGKV3-15	CCL4	IL7R	GNLY	HIST1H4C	GNLY	C1QB
RGS5	CD79A	TCL1A	IGHG1	CCL5	LTB	XCL1	STMN1	NKG7	C1QA
THY1	CD83	MS4A1	IGKV1-5	CD8A	CD2	XCL2	HMGB2	GZMB	C1QC
COL4A1	CD37	CD37	IGKV3-20	CCL4L2	CXCR4	AREG	TYMS	FGFBP2	APOE
COL4A2	BANK1	LINC00926	IGKV3-11	IFNG	TRAC	KLRD1	MKI67	PRF1	APOC1
MYLK	HLA-DRA	HLA-DQB1	IGKV1-9	NKG7	IL32	NKG7	CXCL13	KLRD1	SPP1
SDC2	CD74	HLA-DRA	IGKC	XCL1	CD3E	TRDC	TUBB	GZMH	TYROBP
COL18A1	HLA-DQB1	CD79B	IGLV2-14	GZMA	CD3D	KLRC1	HMGN2	CCL4	FTL
ACTA2	VPREB3	VPREB3	IGKV4-1	CD8B	CREM	GZMB	TUBA1B	GZMA	CD68
BGN	HLA-DQA1	BANK1	IGLV3-25	XCL2	ICOS	CTSW	RRM2	CST7	CXCL8
MYL9	IGHM	IGHD	IGLV1-40	CST7	TNFAIP3	KLRB1	UBE2C	CTSW	CCL3
COL1A1	CD79B	FCER2	IGLV1-44	GZMK	BIRC3	CD7	PTTG1	KLRB1	CTSB
TAGLN	HLA-DPB1	HVCN1	IGHV6-1	ZNF683	KLRB1	HOPX	CENPF	KLRF1	FCER1G
TPM2	HLA-DQA2	HLA-DPB1	IGLC2	CD69	CYTIP	CCL5	CORO1A	MYOM2	LYZ
SPARC	IRF8	ADAM28	IGLV1-51	GZMB	TSC22D3	CD69	NUSAP1	FCGR3A	MS4A6A
CALD1	HLA-DPA1	FCMR	IGHV3-23	GZMH	CCR7	GZMA	TOP2A	PLAC8	HLA-DRA
IGFBP7	ARHGAP24	CD74	IGHV3-21	CTSW	BTG1	IL2RB	HIST1H1E	CCL5	AIF1
TIMP1	ADAM28	LAPTM5	IGLV3-21	RP11-291B21.2	TRBC2	CCL4	PFN1	SPON2	RNASE1
LGALS1	LINC00926	NCF1	IGLC3	CD7	RGS1	DUSP2	HMGB1	HOPX	HLA-DRB1
TUBA1B	IGHA1	CD83	IGLV1-47	CD3E	SPOCK2	CMC1	H2AFZ	CD247	CCL3L3

Monocyte	Cycling_Myeloid	DCs	Myoepithelial	Luminal Progenitors	Mature Luminal	Cancer Cycling	Cancer Her2 SC	Cancer LumB SC	Cancer Basal SC	Cancer LumA SC
LYZ	C1QB	HLA-DPB1	KRT14	KRT15	SCGB3A1	CD24	CD24	SLC39A6	S100A8	SCGB2A2
IL1B	C1QC	HLA-DRA	KRT17	LTF	AZGP1	S100A8	MUCL1	DHRS2	SAA1	SCGB1D2
G0S2	C1QA	HLA-DPA1	MYLK	PTN	PIP	S100A9	CALML5	AGR3	RARRES1	PIP
TYROBP	LYZ	GPR183	KRT5	MMP7	MGP	MUCL1	MIEN1	COX6C	MGST1	ELF3
FCN1	AIF1	HLA-DQA1	TAGLN	MGP	XBP1	STMN1	KRT7	H2AFJ	MGP	SCGB2A1
AIF1	TYROBP	HLA-DQB1	ACTG2	CXCL2	TFF1	S100A2	DBI	AGR2	CALML5	S100A14
PLAUR	FCER1G	CD74	DST	TACSTD2	C8orf4	UBE2C	ERBB2	TSPAN13	KRT7	AGR2
LST1	CD68	HLA-DRB1	ACTA2	SLPI	STC2	MGST1	IDH2	ANKRD30A	EPCAM	AZGP1
FCER1G	MS4A6A	HLA-DRB5	CXCL14	SFRP1	AREG	HIST1H4C	KRT19	STC2	S100A2	KRT18
S100A9	SPP1	CST3	CSRP1	CLDN4	KRT18	KRT7	GRB7	STARD10	CSTB	TFF1
CXCL8	APOC1	IRF8	CNN1	SAA1	KRT8	TUBB	SPINT2	MAGED2	CD24	CLDN4
HLA-DRA	HLA-DRA	PLAC8	C2orf40	TM4SF1	MAFF	TK1	SLPI	MLLT4	PFDN2	S100A1
SERPINA1	HLA-DPA1	LILRA4	FBXO32	CCL28	CLDN4	EPCAM	SERINC2	AZGP1	MARCKSL1	CLDN3
C15orf48	TUBA1B	HLA-DQA2	MT1X	CRYAB	WFDC2	RARRES1	FABP7	XBP1	LDHB	ANKRD30A
HLA-DPB1	HLA-DRB5	LYZ	SFN	WFDC2	TM4SF1	HMGA1	S100A8	KRT19	GAPDH	KRT19
HLA-DPA1	HMGN2	CD83	MT1E	KRT23	EFHD1	IDH2	CLDN3	FXYD3	TPD52L1	CALML5
BCL2A1	GPX1	LGALS2	CRYAB	GABRP	ANKRD30A	CKS1B	S100A9	CRABP2	PERP	EFNA1
MS4A6A	CTSB	IRF7	MT2A	RCAN1	TFF3	CENPF	RP11-206M11.7	ARMT1	CCND1	KRT8
EREG	FTL	GZMB	TPM2	KRT7	SERPINA1	AIM1	KRT8	CCND1	S100A9	MUC1
CXCL2	APOE	AREG	EGR1	NDRG2	MUCL1	CKB	ATG5	MRPS30	S100A6	AGR3

Table S4: EMILIN and clinicopathological factors for 75 breast cancer patients.

Factors	EMILIN Low n=18	EMILIN High n=57	P-value
**Age**			
<60	10	24	0.3176
60≦	8	33	
**T factor**			
T1	9	29	0.9483
T2-4	9	28	
**N factor**			
N0	9	38	0.2025
N1-3	9	19	
**Stage**			
I	6	28	0.2407
II-III	12	29	
**ER**			
Positive	10	29	0.7291
Negative	8	28	
**PgR**			
Positive	5	20	0.5663
Negative	13	37	
**HER2**			
Positive	9	26	0.7451
Negative	9	31	
**Subtype**			
Luminal	4	16	0.8702
Luminal HER2	6	14	
HER2	3	12	
Triple negative	5	15	
**Ki-67 (n=70)***			
< 20%	1	20	0.0126
≥ 20%	16	33	

*Ki-67 data were not available for 5 cases.
